# Biomechanical analysis of an unpowered hip flexion orthosis on individuals with and without multiple sclerosis

**DOI:** 10.1186/s12984-021-00891-7

**Published:** 2021-06-27

**Authors:** Ross M. Neuman, Staci M. Shearin, Karen J. McCain, Nicholas P. Fey

**Affiliations:** 1 Walker Department of Mechanical Engineering, The University of Texas at Austin, 204 E Dean Keeton St, Austin, TX 78712 USA; 2 UT Southwestern Medical Center, 5323 Harry Hines Blvd, Dallas, TX 75390 USA

**Keywords:** Biomechanics, Multiple sclerosis, Hip flexion orthosis

## Abstract

**Background:**

Gait impairment is a common complication of multiple sclerosis (MS). Gait limitations such as limited hip flexion, foot drop, and knee hyperextension often require external devices like crutches, canes, and orthoses. The effects of mobility-assistive technologies (MATs) prescribed to people with MS are not well understood, and current devices do not cater to the specific needs of these individuals. To address this, a passive unilateral hip flexion-assisting orthosis (HFO) was developed that uses resistance bands spanning the hip joint to redirect energy in the gait cycle. The purpose of this study was to investigate the short-term effects of the HFO on gait mechanics and muscle activation for people with and without MS. We hypothesized that (1) hip flexion would increase in the limb wearing the device, and (2) that muscle activity would increase in hip extensors, and decrease in hip flexors and plantar flexors.

**Methods:**

Five healthy subjects and five subjects with MS walked for minute-long sessions with the device using three different levels of band stiffness. We analyzed peak hip flexion and extension angles, lower limb joint work, and muscle activity in eight muscles on the lower limbs and trunk. Single-subjects analysis was used due to inter-subject variability.

**Results:**

For subjects with MS, the HFO caused an increase in peak hip flexion angle and a decrease in peak hip extension angle, confirming our first hypothesis. Healthy subjects showed less pronounced kinematic changes when using the device. Power generated at the hip was increased in most subjects while using the HFO. The second hypothesis was not confirmed, as muscle activity showed inconsistent results, however several subjects demonstrated increased hip extensor and trunk muscle activity with the HFO.

**Conclusions:**

This exploratory study showed that the HFO was well-tolerated by healthy subjects and subjects with MS, and that it promoted more normative kinematics at the hip for those with MS. Future studies with longer exposure to the HFO and personalized assistance parameters are needed to understand the efficacy of the HFO for mobility assistance and rehabilitation for people with MS.

**Supplementary Information:**

The online version contains supplementary material available at 10.1186/s12984-021-00891-7.

## Introduction

Multiple sclerosis (MS) is chronic neurological disorder in which inflammation leads to the demyelination of nerve fibers and the eventual breakdown of neurons in the central nervous system. This damage causes a long-term accumulation of disability, resulting from sensory and motor impairments [1]. In 2015, over 2 million cases were reported globally [2], and an estimated 75% of people with MS experience mobility impairments over the course of their disease [3]. These deficits, often emerging in early adulthood, constrain activities of daily living and appear to negatively affect quality-of-life [4].

MS-related symptoms such as muscle weakness, spasticity, and sensory changes are highly variable across individuals. Symptoms can vary throughout the day due to fatigue, and as the disease progresses, individuals can experience relapses, remissions, and increasing disability [3, 5, 6]. The effects of MS on gait often include reductions in step length, walking speed, dynamic stability, and range of motion (ROM) in lower limb joints [7–10].

To address the various mobility limitations caused by MS, a wide range of devices are prescribed to patients. While severe impairments necessitate wheelchairs, ambulatory people with MS often use ankle–foot orthoses (AFOs), crutches, canes, or walkers. While these are helpful interventions for walking impairments, the variability of gait impairment amongst individuals with MS and the lack of evidence-based practice in prescribing mobility devices to people with MS have led to high rates of abandonment and low satisfaction with this equipment [11, 12]. For instance, a common manifestation of MS is difficulty clearing the foot during the swing phase of gait, which is often attributed to dorsiflexion and eversion weakness in the ankle. AFOs are frequently prescribed in this situation [13, 14]. While the literature has shown that AFOs can produce measurable gait improvements in people recovering from stroke [15–19], the data regarding their effects in the context of MS is sparse and inconclusive [13, 20, 21]. This demonstrates the lack of MS-specific research into mobility interventions, and the need for further development of mobility-assistive technologies (MATs) for people living with neurodegenerative disorders.

In recent years, research in MATs has focused on the development of wearable robotic exosuits and exoskeletons. These devices aim to augment human gait through the controlled actuation of motor-driven cables or pneumatic artificial muscles that span various joints in the lower limbs [22–24], or motors situated concentrically with joints [25, 26]. Studies have demonstrated that such technology can reduce muscle activation during unloaded [23, 27–30] and loaded walking [22, 31], and reduce the metabolic cost of walking in both healthy subjects [23, 27, 32–34] and subjects with post-stroke hemiparesis [35, 36]. While promising, the costs, power demands, environmental adaptability, noisiness, and size of these devices are hurdles that must be overcome for widespread adoption of this technology [37–39].

Despite the practical barriers currently preventing most exosuits from reaching consumers, the research related to these devices has been vital to learning about the biomechanical effects of augmenting forces about the lower-limb joints. Exosuit studies have demonstrated the virtues of carefully-timed assistive forces about the hip during walking [28–31, 33, 34]. Considering the crucial role that hip flexors play in the swing phase of gait [40, 41], the lack of clinically available hip-centric orthoses for mobility assistance suggests a need for more investigation in this area. In 2008, Sutliff et al. showed that a passive hip flexion-assisting orthosis, consisting of a waist belt with resistive components that span the hip and knee joints, improved clinical gait assessment scores in a group of people with MS over a 12-week period [42]. A similar passive device with elastic components spanning only the hip joint produced significant improvements in timed 6 and 10-min walk tests in a group of persons exhibiting hemiparetic gait post-stroke [43]. More recently, Panizzolo et al. found that a bilateral passive hip flexion device reduced net metabolic power compared to free walking in older healthy adults [44]. While these studies show promising results based on clinical and metabolic assessments, biomechanical investigations of passive hip orthoses that include inverse dynamics and muscle activity have not been published.

In this paper, we present a custom passive, lightweight, unilateral hip flexion orthosis (HFO), and investigate its biomechanical and neuromuscular effects on individuals with and without MS. The orthosis consists of elastic bands that span the hip joint, store energy during hip extension in stance, and release the stored energy to assist flexion upon swing initiation. This exploratory study examined the effects of passively augmenting hip flexion in impaired gait, and is an important step toward conducting quantitative biomechanical analyses of novel mobility interventions for people with MS.

The aim of this study was twofold: first, to determine whether people with and without MS can tolerate the HFO in steady-state, level walking; and second, to investigate subjects’ biomechanical and neuromotor responses to the HFO under three different stiffness configurations. While we detail the effects of the device for subjects with and without MS, this study does not attempt to match these two groups. The non-MS group was the initial cohort to test the device, and represents a general control response that can be used as a basis of comparison for further studies involving a variety of pathologies. Testing with the MS group followed the successful completion the non-MS trials, and serves as an exploratory study of the effects of a novel device on people with varying levels of mobility impairment due to a neurological disorder.

We hypothesize that that participants’ peak hip flexion angles will increase in the assisted leg while wearing the HFO, and that muscle activity will increase in hip extensors while wearing the HFO, and decrease in hip flexors and plantar flexors.

## Materials and methods

### Description of the passive unilateral hip flexion orthosis

**Fig. 1:**
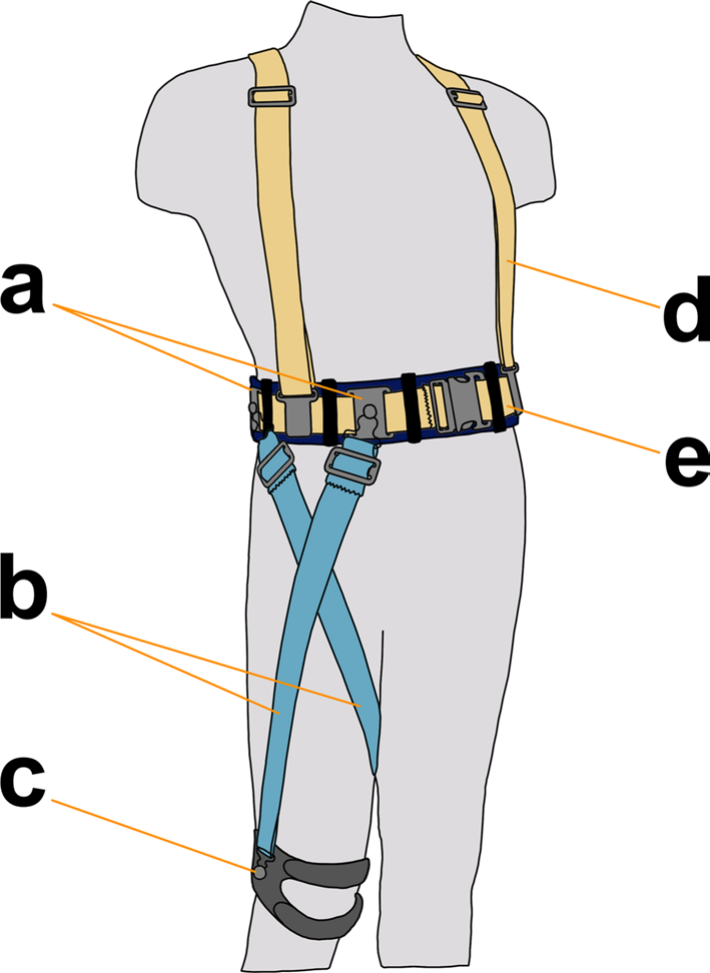
HFO Overview. **a** Waist anchors secure resistance bands to waist belt. Anchor locations can be individually adjusted about the belt. **b** Resistance exercise bands passively store and release energy during gait. **c** Knee brace for distal anchoring of bands. **d** Suspenders distribute load of bands about torso and help support waist belt. **e** Waist belt for proximal anchoring of bands. A soft neoprene base layer provides comfort, while a nylon webbing strap provides rigidity and connects to the waist anchors and suspenders

In this study, our custom hip flexion orthosis is configured for unilateral assistance. The HFO (Fig. 1) consists of: a nylon waist belt for proximal anchoring of resistance bands with a neoprene fabric base layer to interface with the torso; an off-the-shelf neoprene knee brace for distal anchoring of the exercise bands; off-the-shelf elastic suspenders to support the waist belt and distribute tensile forces about the torso; and two resistance exercise bands (TheraBand®) to store and release mechanical energy throughout the gait cycle. The total cost of fabricating the HFO, including custom and off-the-shelf components, was approximately $65.

Each resistance band is tensioned between quick-release attachment points on the waist belt and knee brace, creating a passive flexion moment about the hip joint. The two bands are arranged antagonistically (crossing over at mid-thigh) to aid rotational stability and maintain a low profile. The band that anchors closer to the navel is referred to as the “medial” band, while the band that anchors near the iliac crest is called the “lateral” band. The locations of the waist anchors can be independently adjusted to meet the specific needs of the user.

The HFO is low-profile and it does not extend below the knee, allowing for simultaneous use of an AFO if desired. The HFO is also designed to cater to dexterity limitations experienced by people with MS [3]: when the user assumes a sitting position, tension in the bands is relieved, and the quick-release attachments can be easily connected or disconnected. The waist belt is fastened with a simple buckle, and with the suspenders it can be donned and doffed much like a backpack. The knee brace is fastened with two hook-and-loop straps.

Off-the-shelf resistance exercise bands are used to provide the assistive moment. These bands are commonplace in physical therapy clinics, where the HFO would be configured by the clinician. The wide range of available band stiffnesses provides the ability to adjust the HFO to an appropriate resistive force on a case-by-case basis.

### Experimental design

This study was approved by the UT Dallas Institutional Review Board (MOD 1-CL 17-170), and participants provided written informed consent. Five volunteers with no mobility impairments and five volunteers with unilateral hip flexor weakness due to MS were recruited to wear the device configured in four different conditions: no resistive bands; nominal-stiffness bands (B1); intermediate-stiffness bands (B2); and high-stiffness bands (B3). Participant data can be found in Table 1. The MS participants were recruited from the Gait Disorder Clinic at the UT Southwestern School of Health Professions. Inclusion criteria were a confirmed diagnosis of MS, the ability to walk at least 150 feet without physical assistance (with or without an AFO), unilateral hip flexor weakness of 2+/5 or less as determined by Manual Muscle Testing [45], ages 18–75 years, and body mass index less than 35. Volunteers were excluded if they had other neurologic or orthopedic diagnoses that would negatively impact walking. Participants were encouraged not no rely on the handrails of the treadmill for walking, however light use of the treadmill handrails was permitted. One participant opted to wear an AFO during the trials.

**Table 1:** Participant data

Subject ID	Age	Gender	Height (m)	Mass (kg)	Years with MS	Assisted hip flexor strength	Treadmill velocity (m/s)
MS1	57	F	1.64	88.5	24	2−/5	0.3
MS2	61	F	1.59	51.3	7	2−/5	0.25
MS3	45	F	1.69	56.5	24	1/5	0.2
MS4	54	F	1.6	88.2	5	2+/5	0.4
MS5	52	F	1.58	87.9	2	1+/5	0.2
C01	22	M	1.88	81.6	–	–	1
C02	45	M	1.86	103.6	–	–	1
C03	21	M	1.66	108	–	–	1
C04	58	F	1.7	57.4	–	–	1
C05	35	F	1.76	80.1	–	–	1

Participants had no experience using the HFO prior to data collection. After recording anthropometric data, participants were fitted with the HFO. The leg to which the device was fitted is referred to as the *assisted* leg, and the other the *unassisted* leg. Individuals with MS wore the device on their weaker leg, and control subjects wore the device on their non-dominant leg. The distance between the proximal and distal attachment points for both bands was recorded in a neutral standing position, and band segments were sized to a resting length of 75% of this distance. This pre-tension value was chosen subjectively after preliminary tests found it to produce noticeable sensation in B1 without being prohibitively stiff in B3.

Participants with MS were allowed to self-select a comfortable walking pace under HFO assistance on a treadmill, and all controls walked at 1 m/s. We chose a fixed pace for the control group to allow for closer comparison across joint conditions of the joint angles, moment, and muscle activity, which are known to vary significantly with walking speed [46]. For participants with MS, it was not feasible to fix the pace given the varying functional abilities of the individuals in the group. When ready, trials were conducted wherein the subject was recorded for one minute of steady-state, level walking. First, a no-bands trial (N1) was captured for an initial baseline, followed by the nominal (B1), intermediate (B2), and stiff (B3) conditions, in randomized order. Participants completed further no-bands trials (N2, N3, and N4) following each band condition. An example of the full randomized protocol for a subject would be N1-**B3**-N2-**B1**-N3-**B2**-N4. Participants were allowed as much time as they desired to rest between trials.

### Joint kinetics and kinematics

Three-dimensional kinematics were recorded by a ten-camera motion capture system (Vicon, Oxford, UK) at 100 Hz, and marker tracking was performed using Vicon Nexus. Three-dimensional ground reaction forces (GRFs) were recorded for each leg with an instrumented split-belt treadmill (Bertec, Columbus, OH, USA) at 2000 Hz. GRF and kinematic data were low-pass filtered (4th order Butterworth, 10 Hz cutoff) and inverse dynamics calculations were conducted in Visual3D (C-Motion, Kingston, ON, CA) to estimate joint moments and powers. Time integrals of positive and negative joint powers were calculated in Matlab (MathWorks, Natick, MA, USA) to estimate positive and negative joint work, respectively. The last ten good strides of each trial were used for statistical analysis. One control subject (C03) and one subject with MS (P05) were excluded from kinematic/kinetic analysis due to insufficient motion capture marker tracking.

### Electromyography

Muscle activity was recorded at 2000 Hz with surface electromyography (EMG) sensors (Delsys Inc., Natick, MA, USA). Sensors were placed on the tibialis anterior (TA), gastrocnemius lateralis (GAS), soleus (SOL), rectus femoris (RF), vastus lateralis (VAS), biceps femoris (HAM), abdominal obliques (AB), and latissimus dorsi (LAT). Raw EMG signals were band-pass filtered (4th order Butterworth, 20–450 Hz cutoff), rectified, and then low-pass filtered (4th order Butterworth, 6 Hz cutoff) to obtain a linear envelope. Envelopes were normalized to the average peak amplitude of strides during N1. The mean normalized EMG values of the last ten good strides from each trial were used for statistical analysis. In several cases, EMG sensors made poor contact and were omitted from statistical analysis, and are left blank in the results.

### Statistical analysis

Due to the small sample size and high variability of the functional levels of participants, single-subjects analysis was conducted, as group-level analysis would yield results not representative of any particular population [47, 48]. One-way ANOVAs (α = 0.05) were conducted on each observation of interest for each leg. When significant main effects were observed, post-hoc multiple comparisons using the Tukey–Kramer HSD test statistic were performed. These comparisons include all baseline tests for completeness [49], but only the significant pairwise comparisons between the initial baseline (N1) and band (B1, B2, B3) conditions in the assisted leg are reported here for brevity. Full ANOVA results including effect sizes and results on the non-device leg are reported in the Additional file 1. Statistics were computed in Matlab.

## Results

**Fig. 2:**
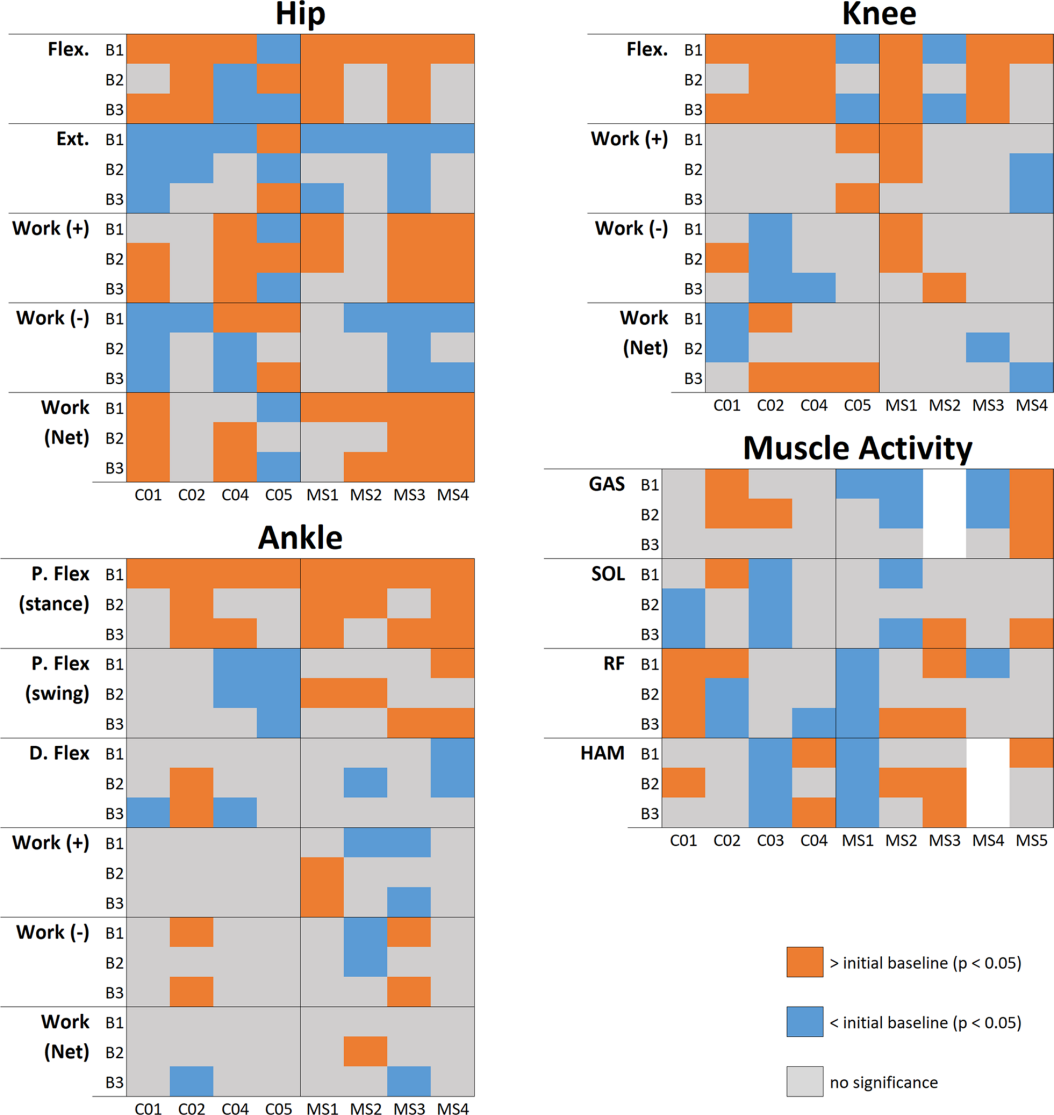
Band conditions resuts overview. Peak flexion and extension angles, average positive, negative, and net work, and average muscle activity. Orange blocks indicate results significantly greater than N1 condition, blue blocks indicate results significantly less than N1, grey blocks represent no significant difference. Empty blocks are due to unusable EMG signals

**Fig. 3:**
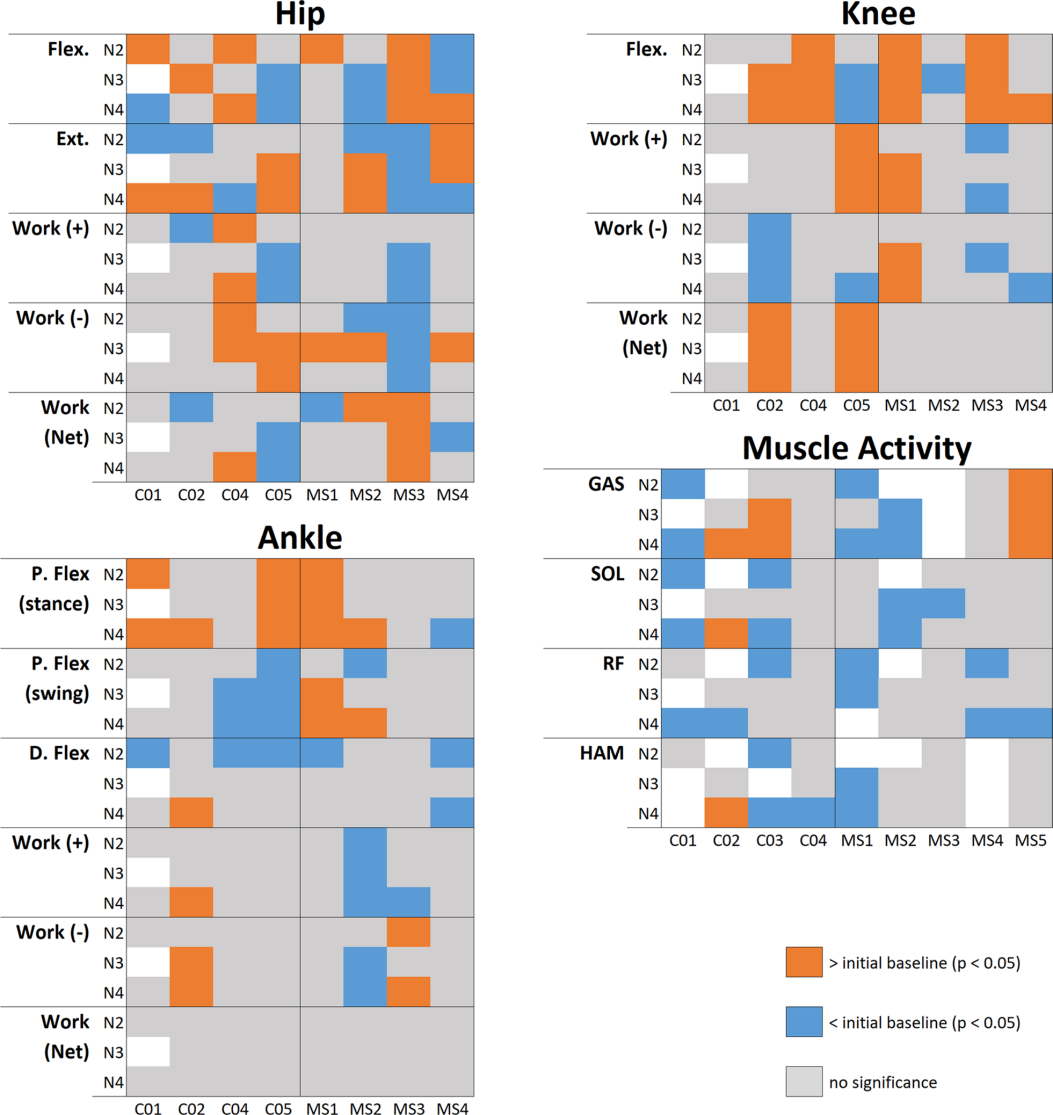
Baseline conditions resuts overview. Peak flexion and extension angles, average positive, negative, and net work, and average muscle activity. Orange blocks indicate results significantly greater than N1 condition, blue blocks indicate results significantly less than N1, grey blocks represent no significant difference. Empty blocks are due to unusable EMG signals

We present a summary of all ANOVA results in Figs. 2 and 3. These figures indicate the significant pairwise results between initial baseline (N1) and subsequent conditions for all subjects. Rather than numerical values, we simply indicate the direction of change, if any, that was observed for a given subject and condition, to aid in identifying trends that may be present. Full numerical results can be viewed in Additional file 1.

### Joint kinematics

**Fig. 4:**
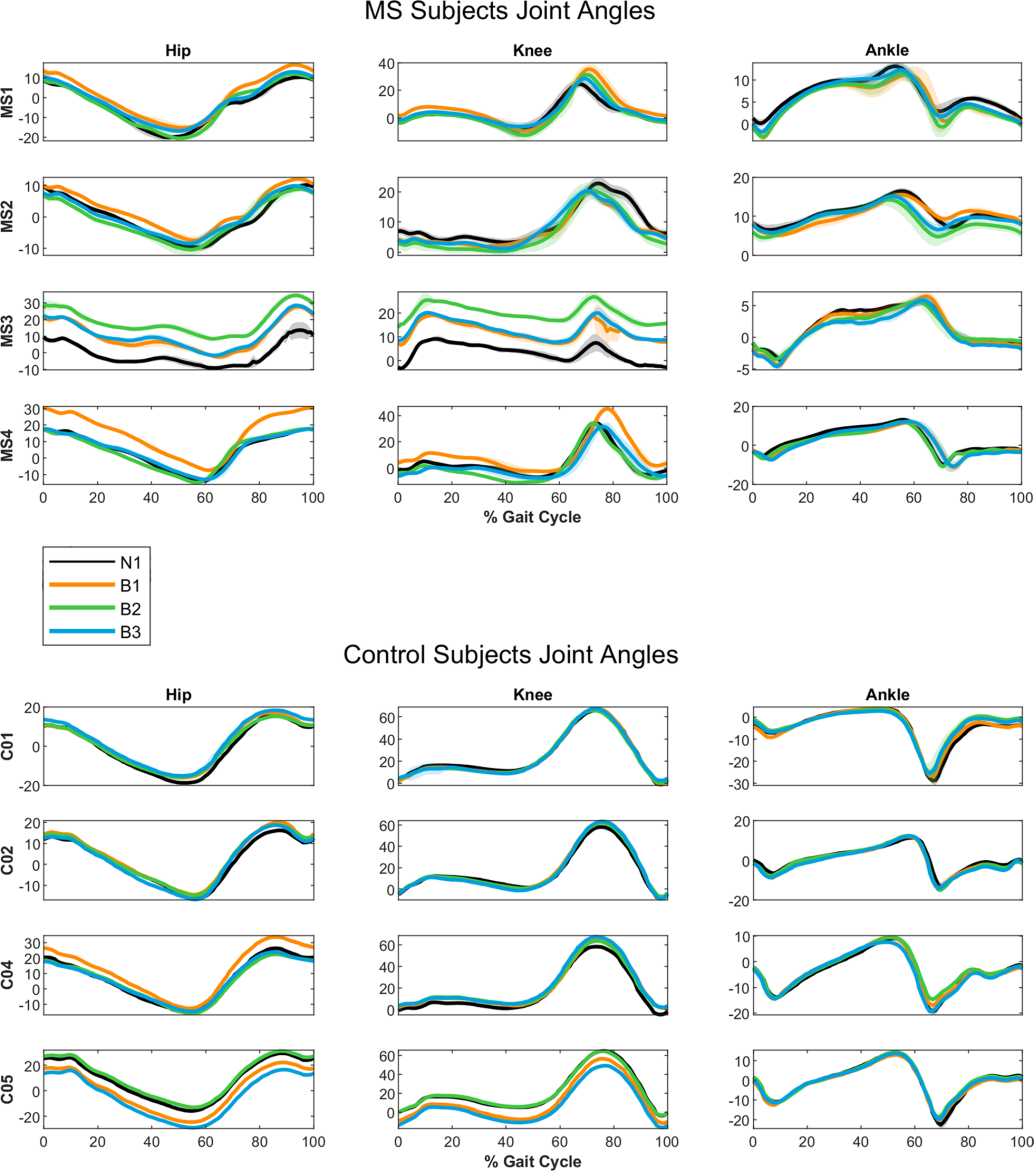
Joint Trajectories. Time-normalized joint trajectories for MS (top) and control (bottom) subjects plotted over the gait cycle for hip, knee, and ankle

Hip kinematics results are reported in Figs. 2 and 3, and averaged individual joint trajectories can be viewed in Fig. 4. In support of our hypothesis, all subjects with MS experienced a significant increase in peak hip flexion angle compared to baseline when using the HFO. While these effects were bilateral in several cases, they were more pronounced in the assisted leg, particularly during the B1 condition. Control subjects also showed increases in hip flexion, though not as drastic as those seen in subjects with MS, and with some band trials producing decreases in peak angle for C04 and C05.

Peak hip extension angles were significantly smaller for most MS and control subjects during HFO trials. Again, the greatest changes in peak angles were typically seen during the B1 condition. Significant results of both increases and decreases in hip kinematics were reported during subsequent baseline tests (N2, N3, N4) for both groups.

An increase in peak knee flexion angle was common, occurring for all subjects except for MS2 and C05, who saw decreases. These trends were observed in baseline and HFO trials.

Stance phase peak plantar flexion increased under at least one band condition for all subjects. Swing phase peak plantar flexion increased under at least one band condition for all of the MS group and decreased for C04 and C05. Peak dorsiflexion was largely unaffected, with primarily decreases observed. For most subjects, ankle kinematics experienced significant changes in both HFO and baseline trials.

### Joint energetics

**Fig. 5:**
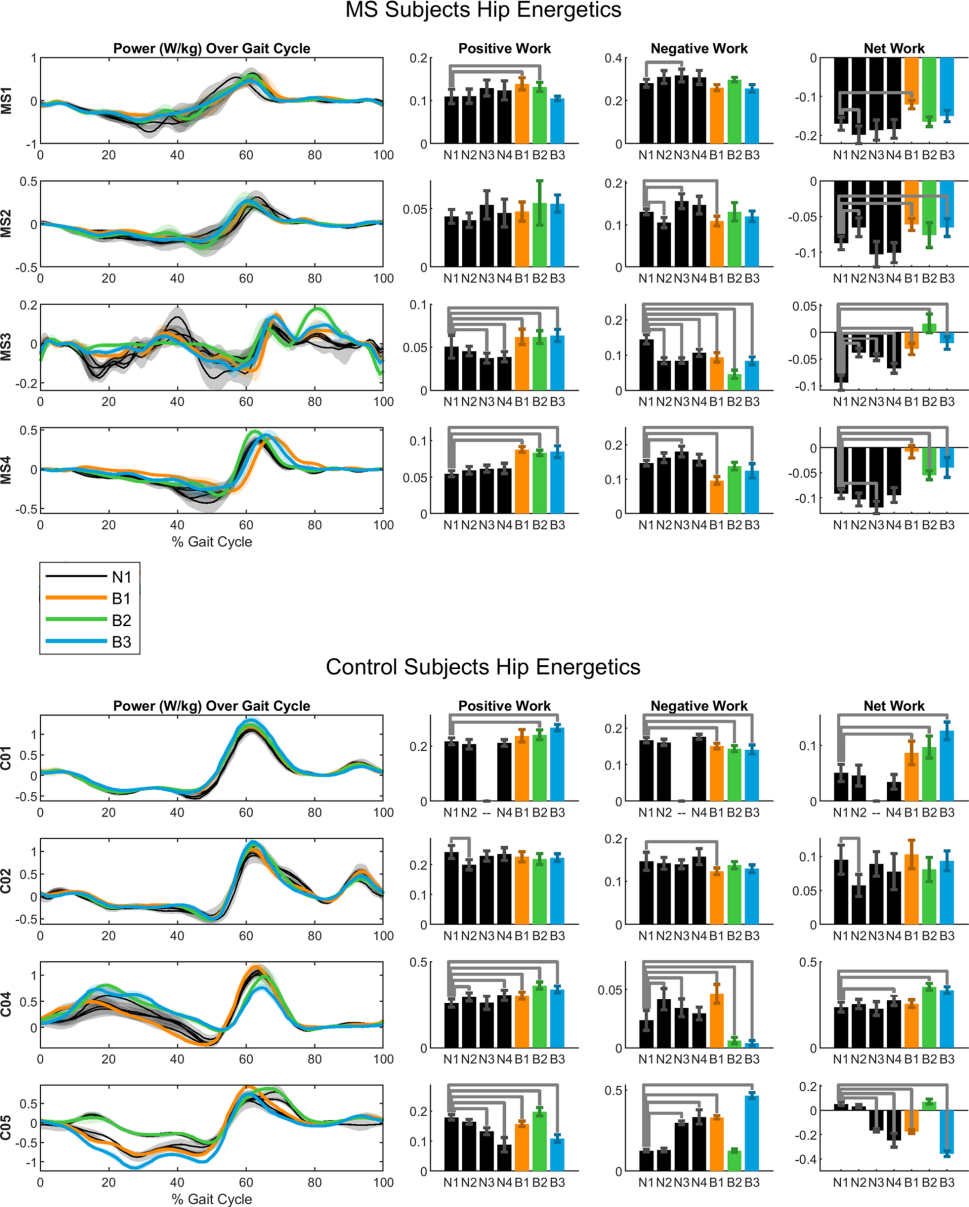
Hip Energetics. Hip power curves over gait cycle and corresponding positive, negative, and net work results for MS group (top) and controls group (bottom)

Positive work in the assisted hip increased during HFO trials for most subjects (MS3 and MS4 for all HFO conditions), but did not increase for these subjects during baseline trials (Fig. 5). Positive work in the unassisted hip was almost completely unaffected. Negative work in the assisted hip either decreased or remained the same for all subjects with MS and for most controls. The resulting net work produced by the assisted hip increased during HFO trials for all subjects with MS.

Work at the knee and ankle was less affected than at the hip, and there was no apparent trend in those results, with varied effects on both baseline and HFO trials.

Peak hip flexion moment on the assisted side significantly decreased for all participants with MS during the B1 condition compared with initial baseline. The unassisted side tended to increase slightly or remain unchanged (for unassisted side results, see Additional file 1). Hip extensor moments were less consistent across subjects, though MS1 and MS2 saw the greatest reduction in assisted-side hip extensor moments in the B1 trial.

### Muscle activity

Significant results in muscle activity are reported in Figs. 2 and 3. We hypothesized that hip extensor (HAM) activity would increase, which was observed in MS2, MS3, MS5, C01, and C04 during HFO trials only. Meanwhile, MS1 and C03 showed decreases in HAM activity. We also hypothesized that hip flexor (RF) activity would be reduced, which was not the case with most of the collected data. A small reduction to RF activity was seen in MS4 B1, and in all band conditions for MS1, though the reductions seen in MS1 were comparable to those seen in their baseline trials. Finally, we hypothesized that plantar flexor (GAS, SOL) muscle activity would be reduced by the HFO. For GAS, only MS4 showed reductions in muscle activity exclusively during HFO trials (B1, B2). For SOL, results were similarly mixed, and no clear trends were observed.

## Discussion

### HFO efficacy

The primary purpose of this study was to evaluate the efficacy of a novel hip flexion orthosis worn by people with and without MS. All participants were able to complete trials for all conditions. The HFO was adjusted to successfully fit a wide range of body sizes, and was well-tolerated by all participants. The benefits of a low-profile, discrete device should not be overlooked; device abandonment due to non-acceptance by the user is common [50], and a lower profile device might not interfere as much with activities of daily living. Furthermore, the inexpensive materials with which the device was built would make it highly accessible to those in search of a walking aid, and easy to maintain given the availability of off-the shelf exercise bands.

### Biomechanical results

**Fig. 6:**
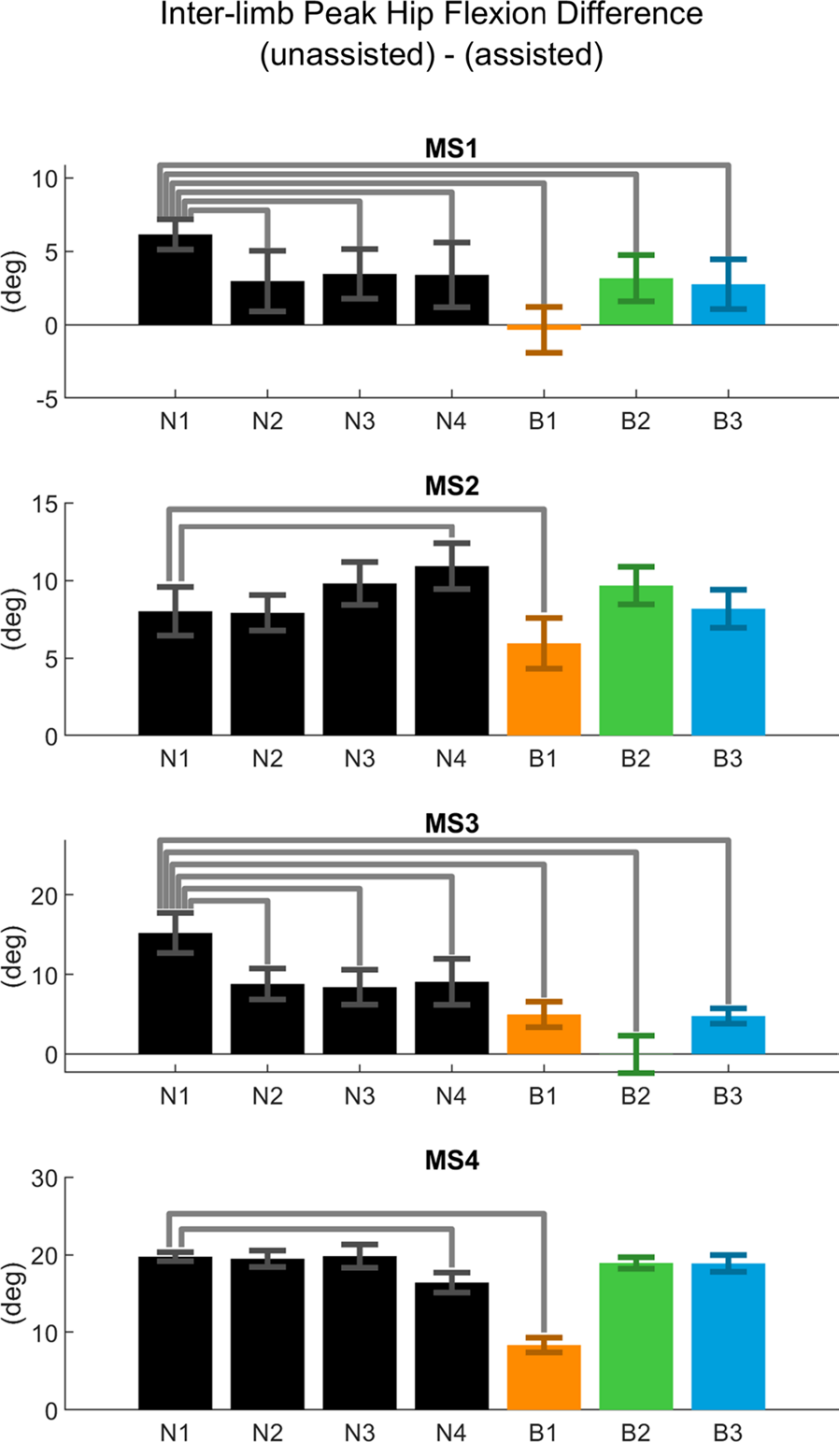
Inter-limb peak hip flexion comparison. Difference in degrees between the unassisted and assisted leg peak hip flexion angle across all trials for MS group. Bars indicate pairwise significant difference from N1 trial

Our hypothesis that hip kinematics in the assisted leg would shift towards flexion was largely supported by subjects with MS and several controls, particularly in the B1 condition. This change opposes the shift toward hip extension during swing that has been observed in people with MS [7]. These effects were more pronounced for subjects with MS**,** indicating that the HFO was able to “target” the pathological gait patterns exhibited in this group. While changes in kinematics do not necessarily reflect changes in energetics [51], the shift to greater hip flexion suggests an increase in foot clearance and lower risk of toe-drag. To highlight this shift toward normative kinematics, an inter-limb comparison of peak hip flexion angle for subjects with MS is given in Fig. 6. The B1 condition resulted in the lowest inter-limb hip angle asymmetry for all but MS3, and was lower than all baseline trials for subjects with MS.

The HFO demonstrated a contribution to positive work at the hip during swing by assisting concentric contraction (Fig. 5). During stance, it contributed to negative work at the hip by opposing extension. It has been shown that human locomotion requires a net positive amount of work, and that in unimpaired level walking, positive work done by the hip is substantially greater than negative work [52]. In this study, all subjects with MS showed the opposite condition, with negative work exceeding positive work at the hip. While wearing the HFO, however, the net work at the hip was more positive than in no-device trials in nearly every case. In the most extreme case (MS4 B2), we observed a shift to net positive work. These findings suggest that the redistribution of mechanical energy by the HFO improved walking for people with MS.

Our hypotheses that hip extensor muscle activity would increase and hip flexor and plantar flexor activity would decrease were not widely supported by the data collected. A major factor in the variability of the muscle activity data was the limited amount of strides analyzed during the relatively short periods of time that data was collected. Furthermore, MS causes the muscles to fatigue quickly, which has a substantial impact on the content of the EMG. Despite limitations, there was a significant increase in HAM activity compared with initial baseline for five of the subjects, including three from the MS group, however MS1 and C03 both saw decreases in HAM activity across conditions.

### Device configuration

The variability seen in neuromuscular responses, including several instances of subjects showing opposite responses to the same conditions, emphasizes the need for highly customizable assistive devices for people with MS. The members of the MS group in this study demonstrated a wide range of functional ability, but all wore the device in the same three configurations for experimental control. The HFO can be configured in numerous ways, and a customized approach with a clinician would be more appropriate to tailor the setup to the needs of a given individual.

Even with such variability, a consistent result was that the B1 condition tended to produce more pronounced effects than the B2 or B3 conditions. This is a particularly noteworthy finding, because B1 had the lowest stiffness of the bands tested, thereby introducing the smallest external loading. This trend was not due to the stiffer bands restricting range of motion at the hip, as the range of hip motion was generally consistent across trials. This suggests that there is an upper limit on device effectiveness as band stiffness increases, and presents the need for exploration of lower-stiffness bands in future studies. It has been shown that there are diminishing returns with increased assistance magnitude for a powered hip exoskeleton [53], as well as with assistance onset timing for the same exoskeleton [33]. A similar phenomenon may be observed in the HFO, because both the magnitude and timing of its energy delivery are a function of the device configuration. Quantifying these parameters is not trivial: band stiffness, pre-tension, anchoring locations, user anthropometrics, device deformation, and individual gait characteristics all play a role in determining their values. Further exploration is required to determine the optimal energy storage characteristics for the passive elements in the HFO, and how to best assess individual needs when setting up the device.

The results of the present study demonstrate that an inexpensive and mechanically passive orthosis can produce significant effects on leg joint kinematics and energetics, and specifically in people with MS. The changes observed in participants with MS trended toward more normative gait kinematics, suggesting the device helped restore some functionality for these individuals.

### Subsequent baseline trials

The N2, N3, and N4 trials were included to provide a baseline between the tests of the three different band stiffnesses, and to monitor the progression of the baseline condition. Inclusion of these trials showed that the initial baseline is subject to change substantially between device trials. Significant changes in subsequent baseline trials could be the result of acclimation to the task, or to carried-over effects of the device itself. In a longer-term study with fewer variables, inclusion of these baseline trials could help understand whether the device promotes motor learning in users, potentially providing rehabilitative benefits.

### Limitations

The outcomes of this experiment are subject to several limitations. Small sample size and single testing session impacts the generalization of these findings. While controlling band stiffness and pre-tension across subjects was part of the design, it meant that individuals were not afforded the opportunity to acclimate to the HFO and make individualized adjustments as needed. Use of the treadmill versus overground walking may have altered the participants’ normal gait characteristics slightly. Treadmill speed was also held constant, so we could not observe the effects on preferred walking speed within subjects. The use of surface EMG sensors meant that muscle activity could not be measured in the iliopsoas, the strongest hip flexor. The highly variable population of subjects prevented meaningful group-level analysis.

## Conclusions

This study presented a soft, passive, unilateral hip flexion orthosis that was well-tolerated by healthy adults and adults with MS under three levels of compliance. For subjects with MS, all device trials showed a statistically significant increase in the peak hip flexion angle of the assisted leg. Net work at the hip was more positive in people with MS when wearing the HFO. Muscle activity responses were highly varied, emphasizing the need for case-by-case adjustments to the device configuration. This study demonstrates the efficacy of the HFO as a mobility-assisting device for people with MS, and motivates the need for further investigation into the effects altering various parameters of the device. More generally, it demonstrates that passive devices can significantly affect walking mechanics, and that patient populations could benefit from redistribution, rather than addition, of mechanical energy from a wearable device.

## Supplementary Information

**Additional file 1**. ANOVA numerical results including effect sizes and results from non-device-wearing leg.

**Additional file 2**. Data from which ANOVAs were carried out in .xlsx format. Includes joint mechanics and muscle activity for both legs of all participants.

## Data Availability

Additional file 1 contains numerical results of the ANOVAs whose significance are reported in Figs. 2 and 3 in table format, as well as the effect size for each test. It is an.xlsx file with four sheets: ankle, knee, hip, and muscle activity. Additional file 2 contains the data from which ANOVAs were carried out. It in an.xlsx file with two sheets: joint mechanics and muscle activity. Each column, beginning at the top, consists of: the participant ID (as used in this manuscript); device or non-device leg; device condition; type of signal (i.e. angle, moment, power, muscle activity) and units; joint or muscle being measured; and 100 data points representing the full stride represented from heel strike to subsequent heel strike.

## Abbreviations

MS: Multiple sclerosis
MAT: Mobility assistive technology
HFO: Hip flexion orthosis
ROM: Range of motion
AFO: Ankle–foot orthosis
GRF: Ground reaction force
EMG: Electromyography
TA: Tibialis anterior
GAS: Gastrocnemius lateralis
SOL: Soleus
RF: Rectus femoris
VAS: Vastis lateralis
HAM: Hamstring (biceps femoris)
AB: Abdominal obliques
LAT: Latissimus dorsi
